# Supplementary material for: Towards a Cytometry Foundation Model: Interpretable Sample-level Predictive Modelling via Pretrained Transformers
Source: bioRxiv. 2026 Apr 2:2026.03.31.712806. Preprint. [Version 1] doi: 10.64898/2026.03.31.712806 (PMC13060299; doi:10.64898/2026.03.31.712806)
Supplement: Supplement 1 [file media-1.pdf]

Supplementary Information for: Towards a Cytometry Foundation Model: Interpretable Sample-level Predictive Modelling via Pretrained Transformers

Zixin Zhuang[1*], Benjamin S. Mashford[2,1], Liang Zheng[1], T. Daniel Andrews[2,1*]

[1]School of Computing, The Australian National University, Canberra, Australia.
[2]John Curtin School of Medical Research, The Australian National University, Canberra, Australia.

*Corresponding author(s). E-mail(s): zora.zhuang@anu.edu.au; dan.andrews@anu.edu.au;

1 Further information on modelling mouse biological sex across Dataset 1 and 2

Dataset 2 is split into a small training set containing 124 samples taken from 4 experimental batches (Dataset 2 training data) , and a large testing set containing the rest of labelled samples, totalling 6668 (Dataset 2 testing data). The Dataset 2 testing data is reserved for testing for all experiments, and the Dataset 2 training data may be provided in unlabelled or labelled format, depending on the setup.

To ensure optimal performance during generalisation, markers specific to the target dataset (Dataset 2) should be excluded if they were not present in the source training set (Experiment B). As these markers were consistently masked during all training phases, the model could not derive useful representations for them. Attempting to utilise their unmasked values during inference (Experiment C) introduces out-of-distribution noise, which significantly degrades performance, often falling below a random baseline, as shown in Figure 2b (1) in the main text.

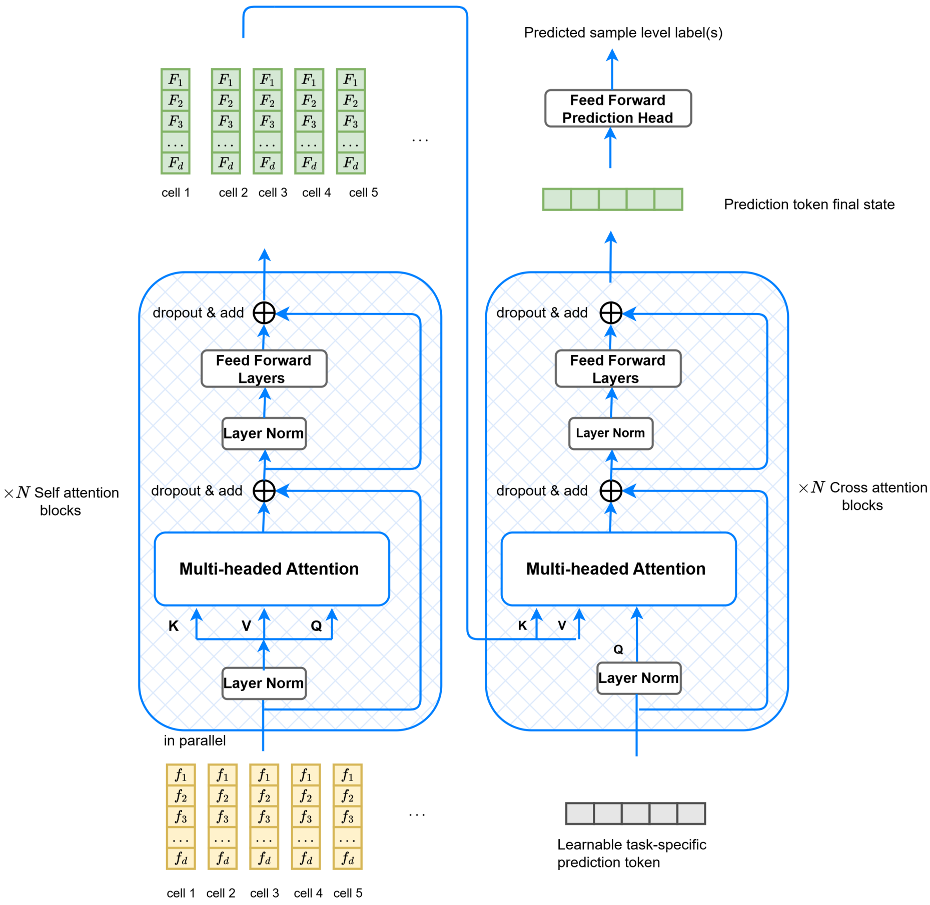

Fig. 1 The Encoder-Decoder architecture of GPCT. No positional encoding is imposed, and layer norms are placed inside the residual connections (Pre-LN style). A learnable task-specific prediction token is utilised by the decoder to produce a sample level prediction.

Table 1 Performance of each model for the gene KO prediction task. Metrics are calculated based on aggregated leave-one-batch-out cross validation results. AUC (ovo) and AUC (ovr) stand for macro-average one-versus-one and macro-average one-versus-rest AUC scores, respectively.

Model	ACC	Macro-average F1	AUC (ovo)	AUC (ovr)
GPCT	**0.694**	**0.683**	**0.903**	**0.901**
GPCT decoder only	0.431	0.427	0.735	0.733
CellCnn	0.458	0.450	0.725	0.726
GPCT generic encoder	***0.736***	***0.737***	***0.921***	***0.919***

Table 2 Marker vocabulary used in this study. Markers in italics are observed in the ENU dataset, bold markers are observed in the KOMP dataset. Bold italics are observed in both.

**Marker**	
ab-TCR	**CD45-2**
**B220**	*CD62L*
**CD1d**	***CD8***
**CD21**	**Foxp3**
**CD23**	*gd-TCR*
CD25	**IgD**
**CD3**	**IgE**
**CD365**	**IgM**
CD4	***KLRG1***
CD44	**NK1-1**
CD45	**CD127**
**CD93**	**CD122**
**CD138**	*CD161*

Table 3 KOMP marker-fluorophore combinations.

**Fluorophore**	**Marker**
APC-A	gd-TCR
APC-Cy7-A	CD8
Alexa Fluor 700-A	CD45
AmCyan-A	CD4
FITC-A	CD44
PE-A	CD25
PE-Cy7-A	KLRG1
PE-Texas Red-A	CD62L
Pacific Blue-A	CD161
PerCP-Cy5-5-A	ab-TCR

Table 4 Shared hyperparameters for GPCT model optimisation, unless otherwise specified. Decoder only variants have reduced dropout ratio to reduce possibility of failure to learn at initialisation.

hyperparameter	default value
batch size	64
optimiser	AdamW
weight decay pretraining	0.005
weight decay downstream	0.001
pretraining learning rate	5e-4
pretraining epochs	300
pretraining cell tokens per sample	5000
pretraining Beta(α_1, β_1)	Beta(1.5,9)
pretraining Beta(α_2, β_2)	Beta(5,16)
pretraining Beta(α_3, β_3)	Beta(5,15)
pretraining $\beta*$	0.1
downstream learning rate	2e-4
downstream epochs	200 (patience 50)
downstream cell tokens per sample	7000
dropout	0.2 (0.1 for decoder only variants)

Table 5 Shared hyperparameters of model architecture for all GPCT models.

hyperparameter	default value
marker vocabulary size	26
hidden layer size	128
encoder layers (if applicable)	4
decoder layers	4
attention heads	4
input projection	[linear(128), relu]
classification head	[256,128,$n\_classes$]
all prediction heads in pretraining	[128 (shared by all heads), 52, 26]

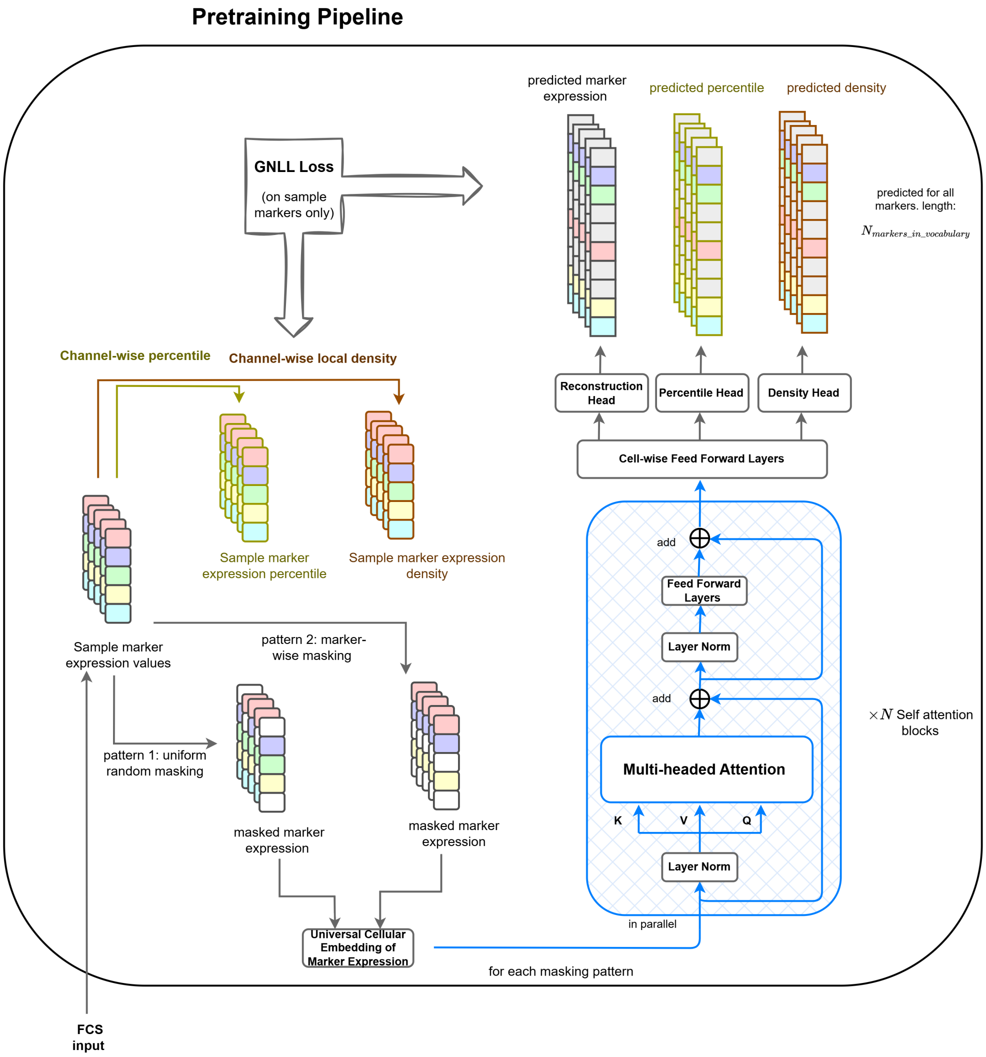

Fig. 2 The self-supervised pretraining pipeline designed for robust cell representation learning: (1) the Percentile and Density matrices are calculated based on raw marker expression (2) The marker expression matrix is masked using two masking patterns, resulting in two masked marker expression matrices. For each masked matrix: (3) The mean and variance predictions of unmasked marker expression, percentile and density matrices for the whole marker vocabulary are made through the [UCEM embedding, GPCT-Encoder, cell-wise prediction heads] pipeline (4) the GNLL loss is calculated for both masked and unmasked entries, but not for initially unmeasured markers (no ground truth available) (5) the loss for the two masking patterns, each with three prediction tasks, are combined using pre-specified weights. Backpropagation is performed on the final loss, and the model parameters are updated.

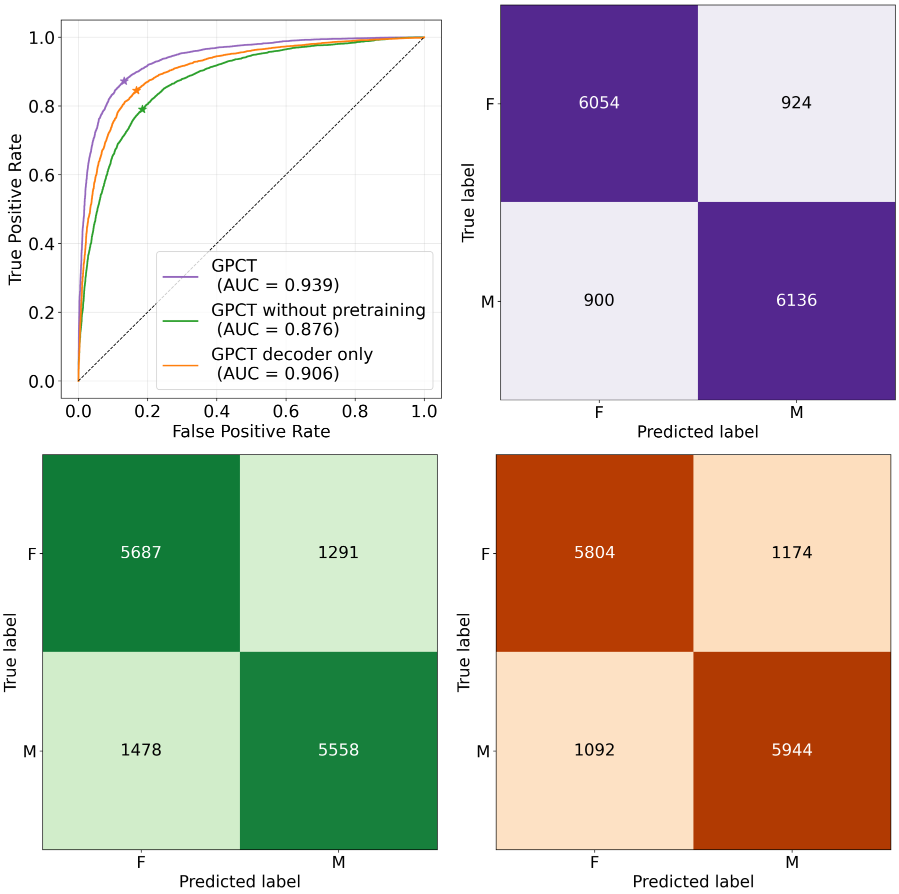

Fig. 3 Results for the ENU mouse biological sex classification: Top left: ROC curves of GPCT, GPCT without pretraining, GPCT decoder only. Top right, bottom left, bottom right: Confusion matrices for GPCT, GPCT without pretraining, and GPCT decoder only. Generated based on aggregated 7-fold cross validation results.

Table 6 Modelling biological sex across KOMP and ENU: Optimisation hyperparameters varied from default values for KOMP-data-only training (KOMP encoder and/or KOMP decoder). KOMP training samples were also upsampled 20 fold when training alongside ENU samples to account for differences in sample support.

hyperparameter	value for KOMP only training
batch size	16
pretraining epochs	2000
downstream epochs	400

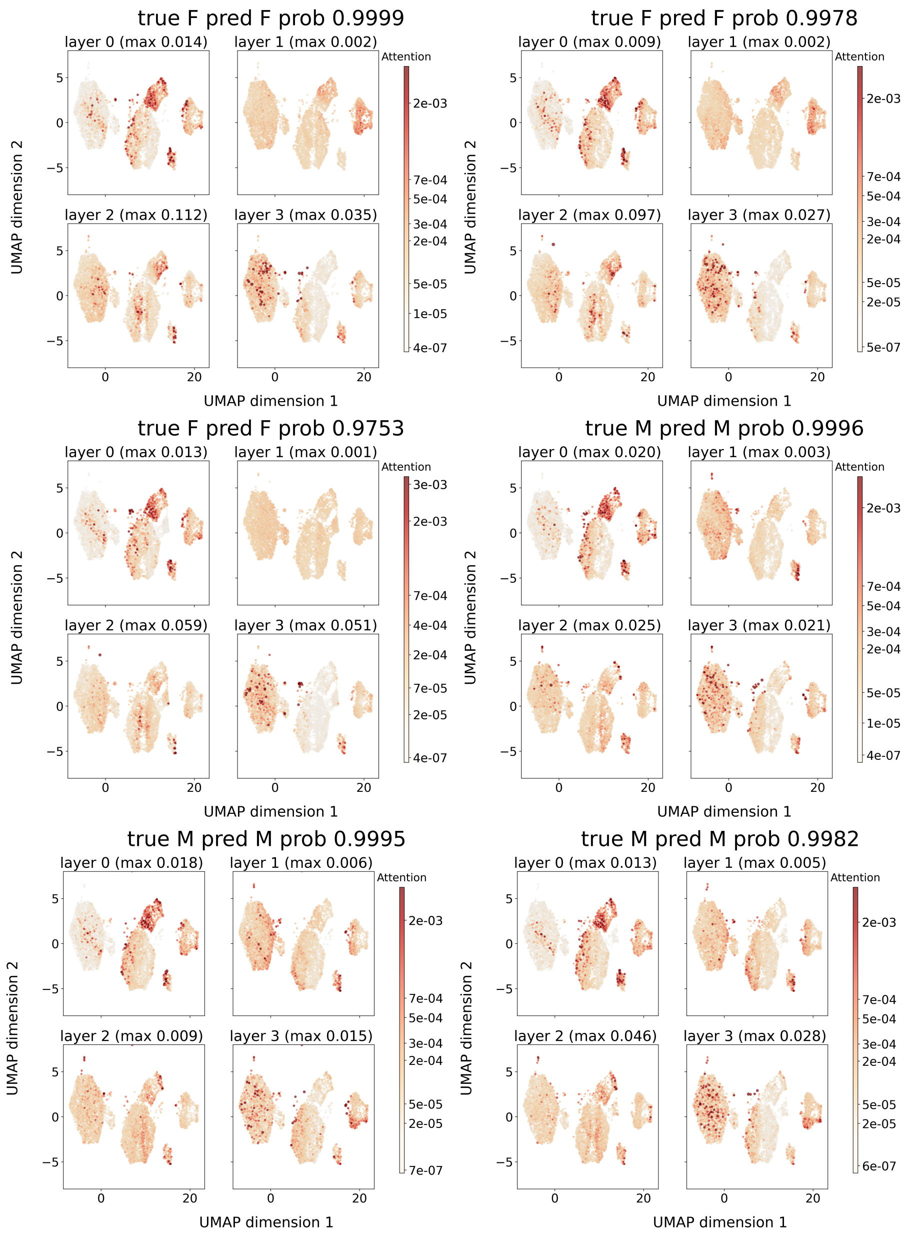

Fig. 4 UMAP visualisation of attention per layer of 6 different ENU mice for the biological sex classification task. The title for each describes the true sex, the predicted sex, and the probability / confidence of the prediction. In general, attention remains consistent across samples of the same sex, and differs slightly for female and male samples, most notably in layer 1.

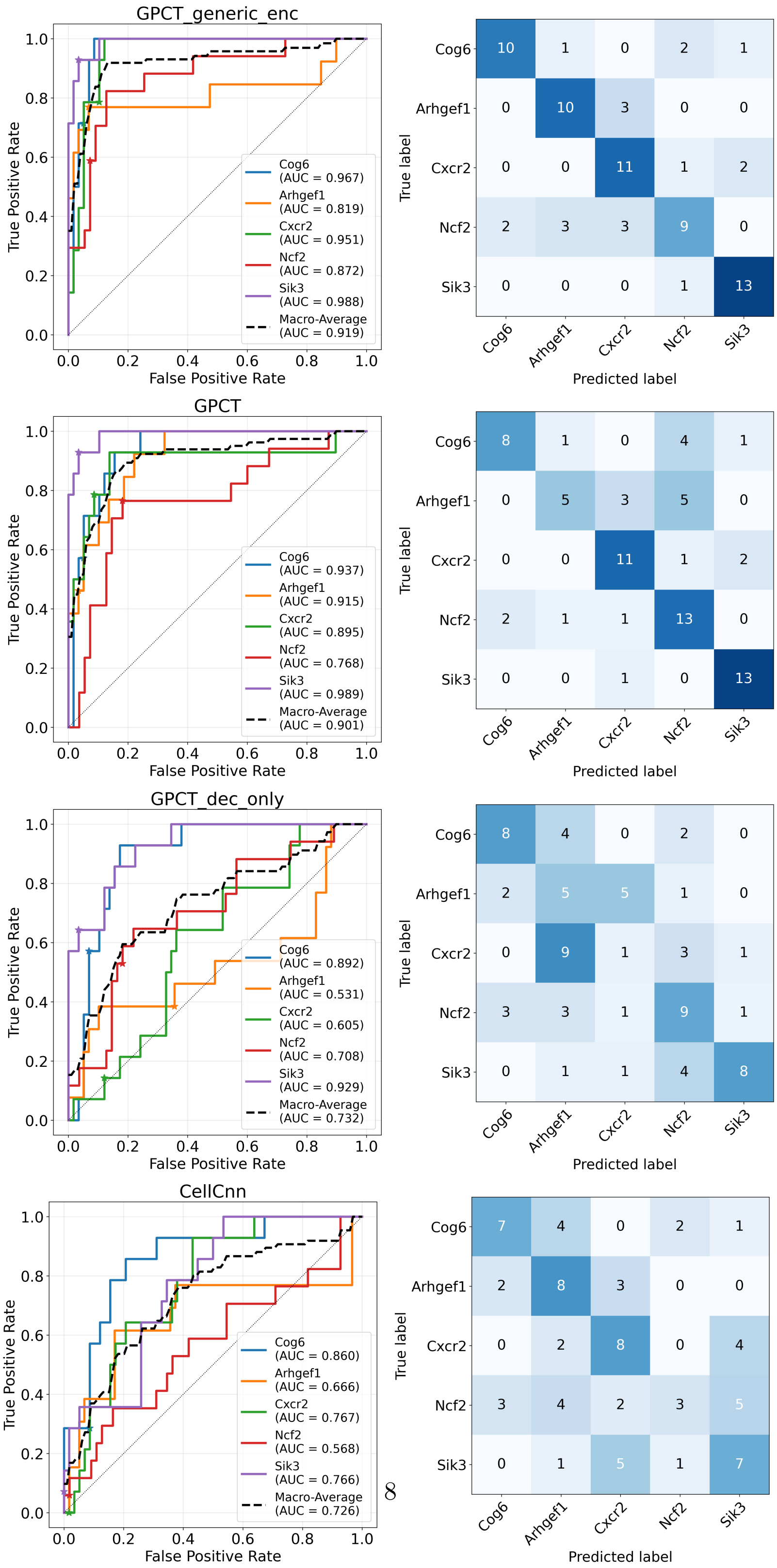

Fig. 5 KOMP gene knockout prediction: The per-class and macro-averaged one-vs-rest ROC curves, as well as the confusion matrix for each of the four models (GPCT generic encoder, GPCT, GPCT decoder only, CellCnn). The star marker denotes the classification threshold of 0.5.

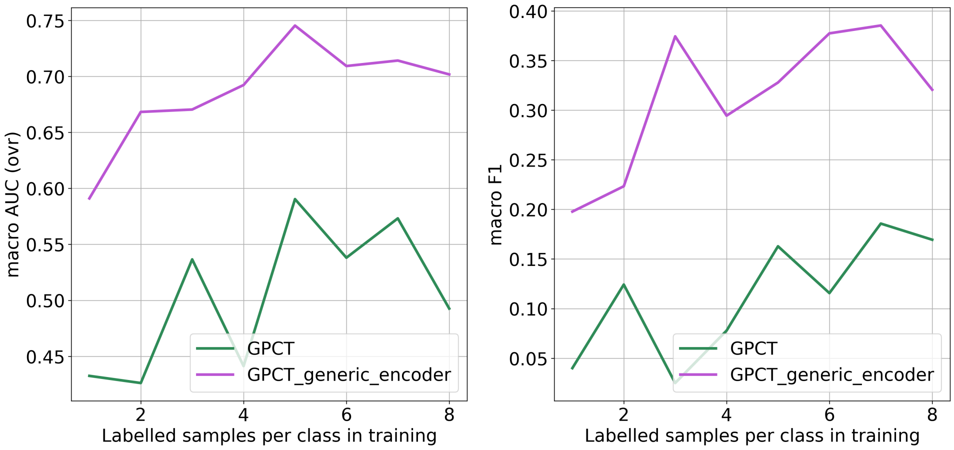

(a) (b)

Fig. 6 Few-shot KO classification experiment on the KOMP dataset, measured by (a) macro-average one-vs-rest AUC and (b) macro-average F1 score. The dataset is split into train ($8 \times 5 = 40$ samples) and test (32) sets, with samples from the same batch kept within the same split to prevent shortcut learning from batch effects. All downstream training ran for 3000 steps at a batch size of 5. Pretraining ran for 6000 steps at a batch size of 5 for GPCT with non-generic encoder, while GPCT with generic encoder used the same generic encoder from the leave-one-batch-out cross validation.

Table 7 Optimisation hyperparameters for KOMP KO predictions that are different from the default, mainly to account for a smaller number of training data. Hyperparameters marked with * do not apply to CellCnn.

hyperparameter	value
batch size	8 (32 for generic encoder)
pretraining epochs	1000 (200 for generic encoder)
weight decay downstream*	0.01
downstream epochs	200 (1000 for decoder only), patience 100
pretraining β*	0.05

Table 8 Distinct marker panels in the ENU dataset and their sample counts

**Marker Panel**	**Count**
B220,CD3,CD4,CD44,IgD,IgM,KLRG1,NK1-1	7002
B220,CD25,CD3,CD4,CD44,IgD,IgM,NK1-1	6013
B220,CD3,CD4,CD44,IgD,IgE,IgM,KLRG1,NK1-1	395
B220,CD3,CD4,CD44,IgD,IgE,IgM	178
B220,CD21,CD23,CD3,CD93,IgD,IgM	122
B220,CD1d,CD3,CD4,CD44,NK1-1	65
B220,CD127,CD3,CD4,CD44,CD8,IgD,IgM	45
B220,CD122,CD127,CD3,CD4,CD44,CD8,KLRG1	44
B220,CD127,CD3,CD4,CD44,CD8	41
B220,CD138,CD3,CD93,IgD,IgM	31
B220,CD138,CD21,CD23,CD3,CD365,CD93,IgD,IgM	27
B220,CD25,CD3,CD4,CD44,CD8,Foxp3	25
B220,CD3,CD4,CD44,CD45-2,IgD,IgM,KLRG1,NK1-1	23
B220,CD25,CD3,CD4,CD44,IgD,IgM	1
B220,CD25,CD3,CD44,IgD,IgM,NK1-1	1
B220,CD3,CD4,CD44,CD45-2,CD8	1
Total	14014

Table 9 Sample support and zygosity for each gene KO class.

Gene	Cog6	Arhgef1	Cxcr2	Ncf2	Sik3	Total
n_samples	14	13	14	17	14	72
Zygosity	Hom	Hom	Het	Hom	Hom	

Table 10: ENU Marker-dye combination counts with marker totals

Marker — Dye Combination	Count	Total for Marker
B220 — APC-A	64	14014
B220 — APC-Alexa 750 / APC-Cy7-A	149	
B220 — FITC-A	25	
B220 — PerCP-Cy5-5-A	13776	
CD122 — FITC-A	44	44
CD127 — PE-A	110	130
CD127 — PerCP-Cy5-5-A	20	
CD138 — PE-A	27	58
CD138 — Qdot 605-A	31	
CD1d — APC-A	65	65
CD21 — PerCP-Cy5-5-A	149	149
CD23 — Alexa 405 / Pac Blue-A	149	149
CD25 — APC-A	25	6040
CD25 — PE-Cy7-A	6015	
CD3 — Alexa Fluor 700-A	197	14014
CD3 — APC-Alexa 750 / APC-Cy7-A	13634	
CD3 — APC-Cy7-A	142	
CD3 — FITC-A	41	
CD365 — Qdot 605-A	27	27
CD4 — Alexa Fluor 700-A	13795	13833
CD4 — APC-Alexa 750 / APC-Cy7-A	17	
CD4 — PE-A	21	
CD44 — Alexa 405 / Pac Blue-A	13489	13834
CD44 — Alexa Fluor 405-A	71	
CD44 — APC-A	178	
CD44 — Pacific Blue-A	71	
CD44 — Qdot 605-A	25	
CD45-2 — APC-A	1	24
CD45-2 — Qdot 605-A	23	
CD8 — APC-A	66	156
CD8 — APC-Alexa 750 / APC-Cy7-A	20	
CD8 — PE-Cy7-A	26	
CD8 — PerCP-Cy5-5-A	44	
CD93 — APC-A	180	180

Continued on next page...

Table 10 – continued

**Marker — Dye Combination**	**Count**	**Total for Marker**
Foxp3 — Alexa 405 / Pac Blue-A	25	25
IgD — FITC-A	225	13838
IgD — PE-A	13613	
IgE — Qdot 605-A	573	573
IgM — FITC-A	13613	13838
IgM — PE-A	31	
IgM — PE-Cy7-A	194	
KLRG1 — PE-Cy7-A	7464	7464
NK1-1 — APC-A	13434	13499
NK1-1 — FITC-A	44	
NK1-1 — PE-Cy7-A	21	
